# Investigation of haptoglobin, serum amyloid A, and some biochemical parameters in calves with omphalitis

**DOI:** 10.14202/vetworld.2018.1055-1058

**Published:** 2018-08-04

**Authors:** K. Bozukluhan, O. Merhan, M. Ogun, B. Kurt, M. Cihan, E. E. Erkilic, G. Gokce, U. Aydin, A. Ozcan

**Affiliations:** 1 Kars School of Higher Vocational Education, University of Kafkas, Kars, Turkey; 2 Department of Biochemistry, Faculty of Veterinary Medicine, University of Kafkas, Kars, Turkey; 3 Department of Surgery, Faculty of Veterinary Medicine, University of Kafkas, Kars, Turkey; 4 Department of Internal Medicine, Faculty of Veterinary Medicine, University of Kafkas, Kars, Turkey; 5 Department of Medical Biochemistry, Faculty of Medicine, University of Kafkas, Kars, Turkey

**Keywords:** acute phase protein, biochemistry parameters, calves, omphalitis

## Abstract

**Aim:**

In this study, it was aimed to determine the concentration of some important acute phase proteins (APPs) and some biochemical parameters pre-operative and post-operative in calves with omphalitis.

**Materials and Methods:**

A total of 20 calves were used in the study and they consist of 10 clinically healthy calves that were used as a control and 10 calves with omphalitis were used as the treatment group. Blood samples were collected from *Vena jugularis* of animals to tubes with anticoagulant (sodium citrate) and without anticoagulants, pre-operative (day 0), and post-operative (day 7). Samples were used to determine the concentration of haptoglobin (Hp), serum amyloid A (SAA), ceruloplasmin (Cp), fibrinogen, glucose, total protein, albumin, urea, total bilirubin, creatinine, calcium, phosphorus, aspartate aminotransferase (AST), alkaline phosphatase (ALP), and gamma-glutamyl transferase (GGT) concentrations.

**Results:**

While the Hp, SAA, Cp, fibrinogen, urea, creatinine, total bilirubin, ALP, and GGT concentrations were statistically and significantly increasing rather than the control group during the pre-operative period for calves with omphalitis, they decreased to the post-operative period. Moreover, an insignificant increase in the glucose, total protein, and AST concentrations and an insignificant decrease in the albumin, calcium, and phosphorus concentrations were statistically determined.

**Conclusion:**

We have the opinion that the assessment of biochemical parameters and especially APP levels in calves with the omphalitis together with the clinical findings may be important in terms of the treatment and prognosis.

## Introduction

The umbilical tissue inflammation (omphalitis, omphalophlebitis, omphaloarteritis, and omphalophlebitis-arteritis) is a problem frequently encountered with calves by having an important place in the umbilical lesion: (i) Not cutting the umbilical cord in hygienic conditions and adequate lengths in the postnatal period, (ii) not being the shelter environment hygienic, (iii) not taking the colostrums inadequate amount, and (iv) being the pathogens present in environment may be considered between the reasons of this inflammation occurring in the umbilical tissue [1,2].

The acute phase response (APR) is a non-specific reaction, shown up in a short time following the tissue damage. It occurs due to the reasons such as inflammation, tissue damage, infection, and immunologic disorders. The most important feature of APR is to cause the production of acute phase proteins (APPs) in liver [3,4]. The APPs are used to determine and diagnose the diseases, follow-up the treatment, specify the patient’s prognosis, and state the non-infectious conditions such as stress [5]. The important APPs for calves are haptoglobin (Hp), serum amyloid A (SAA), fibrinogen, and albumin [6].

As the changes in APP concentration give information about the intensity of inflammatory reaction occur, the aim of the study was to determine the concentration of calves having the umbilical inflammation and reveal their diagnostic and prognostic significance.

## Materials and Methods

### Ethical approval

The research work was carried out with the approval of the Institutional Ethics committee of Mehmet Akif Ersoy University, Faculty of Veterinary Medicine (MAKU 2014/51).

### Animals

In the study, 20 (7 days-1 month age) Brown Swiss calves were used, and they consisted of 10 clinically and healthy calves as a control group and 10 calves with omphalitis as a treatment group. The anamnesis information and the environment in which calves are sheltered after the birth, whether the umbilical care is made, and colostrums are given were learned. The type of umbilical region lesions of calves was determined according to the anamnesis, clinical, and operation results. The blood was centrifuged by taking blood preoperatively (day 0) and postoperatively (day 7) from *Vena* *jugularis* of animals to tubes with anticoagulant (sodium citrate) and without anticoagulants.

### Biochemical analysis

While the glucose, total protein, albumin, urea, total bilirubin, creatinine, calcium, phosphorus, aspartate aminotransferase (AST), alkaline phosphatase (ALP), and gamma-glutamyl transferase (GGT) concentrations were determined using the commercial kits (IBL, Turkey), and the Hp and SAA concentrations were colorimetrically found according to ELISA kits (Tridelta Development Limited, Ireland). Ceruloplasmin (Cp) concentrations were determined according to the spectrophotometric method developed by Colombo and Ricterich [7] (Epoch, BioTek, USA), while plasma fibrinogen concentrations were determined by methods of Millar *et al*. [8].

### Statistical analysis

The SPSS [9] for Windows 20.0 was used in the assessment of study data. The groups’ normal distribution was evaluated by performing Kolmogorov–Smirnov test. Comparison of the independent groups showing a normal distribution was performed by the paired t-test.

## Results

The fever, inappetence, umbilical swelling, pain, temperature, thickening in the umbilical vena, and extension in the craniodorsal direction were determined during the clinical examination of calves with omphalitis. In the operation findings, it was also found that the umbilical vena thickened in different dimensions, cleaved into the surrounding tissues, and thickening progressed up to the liver.

While the Hp, SAA, Cp, fibrinogen, urea, creatinine, total bilirubin, ALP, and GGT concentrations were statistically and significantly increasing rather than the control group during the pre-operative period for calves with omphalitis, they decreased to the post-operative period. Moreover, an insignificant increase in the glucose, total protein, and AST concentrations and an insignificant decrease in the albumin, calcium, and phosphorus concentrations were statistically determined (Table-1).

**Table-1:** Mean values and standard errors of acute phase protein and biochemical parameters in the calves with omphalitis (X±SEM).

Parameters	Control (n=10)	Pre-operative (n=10)	Post-operative (n=10)	p-value
Haptoglobin (g/L)	0.66±0.02^a^	0.210±0.04^c^	0.128±0.03^b^	p<0.01
Serum amyloid A (µg/mL)	18.79±2.66^a^	84.31±11.10^c^	56.82±7.59^b^	p<0.01
Ceruloplasmin (mg/dL)	11.09±1.18^a^	16.55±2.73^c^	13.75±2.36^b^	p<0.05
Fibrinogen (mg/dL)	342.58±42.32^a^	504.36±71.58^b^	392.42±50.06^a^	p<0.01
Albumin (g/dL)	3.22±0.72^a^	2.62±0.51^a^	2.78±0.58^a^	NS
Glucose (mg/dL)	56.11±3.35^a^	66.19±6.29^b^	62.79±6.11^b^	NS
Calcium (mg/dL)	9.92±1.91^a^	7.72±0.46^b^	7.77±0.80^b^	NS
Phosphorus (mg/dL)	6.14±1.19^a^	5.54±1.08^a^	5.51±0.74^a^	NS
AST (U/L)	46.50±7.73^a^	76.18±14,52^b^	66.69±7,50^b^	NS
ALP (U/L)	57.27±8.30^a^	99.05±12.38^c^	82.66±12.18^b^	p<0.01
GGT (U/L)	14.96±3.89^a^	35.00±9.15^c^	26.40±3.82^b^	p<0.05
Urea (mmol/L)	7.17±1.04^a^	47.81±8.95^c^	32.23±8.66^b^	p<0.01
Creatinine (µmol/L)	68.77±9.71^a^	177.40±16.54^c^	125.88±15.13^b^	p<0.01
Total protein (g/dL)	7.43±1.02^a^	8.44±1.12^a^	8.22±1.17^a^	NS
Total bilirubin (µmol/L)	2.81±0.52^a^	12.52±1.15^c^	8.68±1.23^b^	p<0.01

^a, b, c^The groups in the same line labeled different letters are statistically significant (p<0.05, *P<*0.01). NS=Nonsignificant. SEM=Standard error mean, AST=Aspartate aminotransferase, ALP=Alkaline phosphatase, GGT=Gammaglutamyltransferase

## Discussion

The umbilical lesions that develop in the neonatal period are important, causing significant economic losses in calves [1,10]. The umbilical infections develop as a result of not being enforced of the hygiene rules and given of the colostrums sufficiently to calves. In the study, it was stated that the postnatal umbilical care of calves was not made in the neonatal period, hygiene was not paid attention in sheltering places, and colostrums were insufficiently given during the anamnesis received from the animal owners. These factors were specified that they played a key role in the umbilical infection formation and were compatible with the studies performed [11]. In the study, the swelling in the umbilical region of calves with omphalitis, temperature, pain, thickening in the umbilical vena, and extension in the craniodorsal direction was found, as specified in the literature [2,12,13].

Factors such as not cutting the umbilical cord in hygienic conditions, and adequate lengths in the postnatal period, and the presence of pathogens in the environment cause umbilical tissue inflammation [1,10]. In addition, the progression of an infection’s effect on the internal organs in omphalitis causes a decrease in the body resistance and increases in the effective virulence. In conclusion, the formation of severe complications such as septicemia, pneumonia, and enteritis was stated to occur [11]. The inflammation synthesizes the APPs in increasing or decreasing concentrations of the infection and tissue damage. In the study, the Hp, SAA, fibrinogen, and Cp concentrations were determined to increase among the APPs, as they are important for ruminants. The Hp is not found in the serum of healthy calves or found in very few levels (<0.1 g/L) [5,6]. The concentration was stated to increase for many natural and experimental (bacterial, viral, and parasitic) diseases [14-22]. It was also stated that the Hp level may be used in determining an animal’s prognosis, and the prognosis was good when its level was between 0.1 and 1 g/L and bad when it was more than 1 g/L in calves [23]. The SAA, one of the α-globulins, is used to determine the inflammatory events’ activity, follow-up the disease’s course, and assess the treatment’s success administered [24]. It was reported that the Hp and SAA concentrations increased depending on the tissue damage and inflammation occurring in calves with omphalitis, and the prognosis was good due to being the Hp level approximately 0.2 g/L.

The fibrinogen is an important APP for determining the fibrinogen inflammatory response, as it is normally found in the plasma and its level increases in the infectious, purulent, traumatic, and neoplastic diseases [23,25]. In the study performed, the fibrinogen level increased depending on the tissue damage dimension and inflammation.

While the Cp is less frequently being used rather than the Hp, SAA, and fibrinogen in calves, the Cp [26] has an impact on the defense system cells and increases their phagocytosis and antimicrobial powers, by having an adequate care [27]. The reason for this increase may result from its effect on the defense system. The Hp, SAA, fibrinogen, and Cp concentrations postoperatively decreased due to healing the wound on the day 7 and ending the APR.

The ALP concentration, used in researching of the liver and bone diseases, is stated to increase if there are calves having cholestasis, bone degradation, and hepatobiliary circulatory disorders and in case of the glucocorticoids (endogenous/exogenous) and stress. Moreover, while the GGT, one of the specific enzymes of the liver diseases, is almost being found in all tissues, its highest concentration is seen in the kidney and liver. Therefore, the GGT concentration was specified to increase to the cholestasis, liver damage, and kidney insufficiencies [25,28]. The ALP and GGT concentrations increased in the study, and the liver damages occurring in calves with omphalitis are considered to depend on the glucocorticoid activity and/or stress resulting from the disease, according to these results. The total bilirubin concentration was stated to increase the hemolysis, liver damage, and cholecystitis [25]. The total bilirubin concentration increased to the study, and this increase may be related to the liver damages occurred. The urea and creatinine levels [25,28], the indicators of kidney function disorder, rise due to the increase in the protein catabolism occurring in case of the infection and inappetence [29]. A significant increase was determined in the urea and creatinine levels in calves with omphalitis, and the reason for this increase may be probably an increase in the protein catabolism in case of the infection and inappetence. The biochemical parameters including ALP and GGT concentrations, total bilirubin, urea, and creatinine postoperatively decreased on day 7.

## Conclusion

We have the opinion that the assessment of biochemical parameters and especially APP levels in calves with the omphalitis together with the clinical findings may be important in terms of the treatment and prognosis.

## Authors’ Contributions

This work was carried out in collaboration between all authors. KB, OM, and MC: Designed the experimental procedures. KB, OM, and GG: Conducted the research work. OM, MO, and AO: Helped in laboratory analysis. BK, OM, UA, and EEE: Prepared figures, tables, revised, and submitted the manuscript. All authors read and approved the final manuscript.
